# “Silent” kidney stones in “asymptomatic” primary hyperparathyroidism—a comparison of multidetector computed tomography and ultrasound

**DOI:** 10.1007/s00423-016-1520-2

**Published:** 2016-10-12

**Authors:** Andreas Selberherr, Marcus Hörmann, Gerhard Prager, Philipp Riss, Christian Scheuba, Bruno Niederle

**Affiliations:** 1 Section “Endocrine Surgery”, Division of General Surgery, Department of Surgery, Medical University, Währinger Gürtel 18-20, A-1090 Vienna, Austria; 2 Department of Radiology, Medical University, Währinger Gürtel 18-20, A-1090 Vienna, Austria

**Keywords:** Asymptomatic hyperparathyroidism, Kidney stones, Primary hyperparathyroidism, PHPT

## Abstract

**Purpose:**

The purpose of this study was to demonstrate the high number of kidney stones in primary hyperparathyroidism (PHPT) and the low number of in fact “asymptomatic” patients.

**Methods:**

Forty patients with PHPT (28 female, 12 male; median age 58 (range 33–80) years; interquartile range 17 years [51–68]) without known symptoms of kidney stones prospectively underwent multidetector computed tomography (MDCT) and ultrasound (US) examinations of the urinary tract prior to parathyroid surgery. Images were evaluated for the presence and absence of stones, as well as for the number of stones and sizes in the long axis. The MDCT and US examinations were interpreted by two experienced radiologists who were blinded to all clinical and biochemical data. Statistical analysis was performed using the Wilcoxon signed-rank test.

**Results:**

US revealed a total of 4 kidney stones in 4 (10 %) of 40 patients (median size 6.5 mm, interquartile range 11.5 mm). MDCT showed a total of 41 stones (median size was 3 mm, interquartile range 2.25 mm) in 15 (38 %) of 40 patients. The number of kidney stones detected with MDCT was significantly higher compared to US (*p* = 0.00124).

**Conclusions:**

MDCT is a highly sensitive method for the detection of “silent” kidney stones in patients with PHPT. By widely applying this method, the number of asymptomatic courses of PHPT may be substantially reduced. MDCT should be used primarily to detect kidney stones in PHPT and to exclude asymptomatic PHPT.

## Introduction

Asymptomatic primary hyperparathyroidism (PHPT) is defined as biochemically verified PHPT that lacks specific symptoms or signs traditionally associated with hypercalcemia or parathyroid hormone excess [1]. Therefore, patients with PHPT and kidney stone disease are symptomatic by definition. Those patients are at a 15 to 30 % higher risk to develop kidney stones than the general population, in which an incidence of 1 % is described [2–6]. A valid detection tool for kidney stones is crucial at the initial diagnosis of PHPT—especially in patients with mildly elevated laboratory findings [7, 8]—and in the course of follow-up to detect patients with symptomatic disease. The only cure then is parathyroidectomy with a restoration of normocalcemia which, in the majority of patients, results in a resolution of organic manifestation [9]. Nevertheless, postoperative persistent and recurrent stone disease has been reported in up to 17 % in long-term follow-up [2, 9–11]. Calculi burden and new stone formation is important in the clinical evaluation of patients with kidney stone disease in PHPT [12]. Thus, the most sensitive method for evaluation of kidney stones has to be used to reveal persistent or recurrent (newly formed) kidney stones after successful parathyroid surgery [13–15].

The current guidelines on diagnosis and treatment of asymptomatic PHPT [1] recommend evaluation of kidney stone disease and nephrocalcinosis with either abdominal x-ray, ultrasound or computed tomography as equivalent modalities to differentiate between the asymptomatic and the classically symptomatic variant. Ultrasound (US) is continuously performed in the search of kidney stones despite such limiting factors as overlying bowel gas [16]. Non-enhanced helical computed tomography (CT) is a widespread and established method in the detection of kidney stones in patients with acute flank pain [16–18]. With an increase in the use of CT intravenous pyelography, US is currently playing a secondary role in the evaluation of kidney stones [19].

We hypothesize that, in patients with PHPT CT may be more accurate in the detection of kidney stones than US [16]. The aim of the present study was to compare the value of US and CT in detecting silent kidney stones, to identify patients with symptomatic PHPT, to improve the postoperative follow-up of stone carriers and to prevent deterioration of kidney function in patients with PHPT.

## Materials and methods

### Patients

In this prospective study, 40 patients (28 female, 12 male) with biochemically proven PHPT were consecutively evaluated over a 1-year period (see Table 1).

**Table 1:** Laboratory findings (mean ± standard deviation) and demographic data (median age)

	sCa	iPTH	25-OH VitD	1, 25-OH VitD	Crea	P	24 h uCa	*n*	Age	Female	Male
Normal range	2.1–2.6	15–65	> 75	25–86.5	0.5–1.3	0.8–1.6	2.5–7 .5				
Unit	mmol/l	pg/ml	nmol/l	pg/ml	mg/dl	mmol/l	mmol/24 h				
Total	2.783 ± 0.22	191 ± 165	42 ± 30	56 ± 26	0.97 ± 0.23	0.74 ± 0.19	7.9 ± 4.7	40	58	28	12
US positive	2.69 ± 0.14	94 ± 18	43 ± 25	36 ± 13	0.90 ± 0.11	0.69 ± 0.14	7.1 ± 3.4	4	55	3	1
US negative	2.85 ± 0.22	202 ± 170	42 ± 31	59 ± 26	0.97 ± 0.24	0.74 ± 0.19	8.1 ± 5.1	36	60	25	11
MDCT postitive	2.83 ± 0.22	243 ± 215	47 ± 43	50 ± 26	1.01 ± 0.29	0.72 ± 0.12	7.6 ± 2.9	15	56	10	5
MDCT negative	2.83 ± 0.22	160 ± 120	39 ± 19	60 ± 25	0.94 ± 0.18	0.75 ± 0.22	8.2 ± 6.4	25	60	18	7

*sCa* serum calcium; albumine corrected; chemical autoanalyzer (Olympus, Hamburg, Germany), *iPTH* intact parathyroid hormone (Elecsys 1010 Autoanalyzer (Roche, Mannheim, Germany), *25-(OH)D3* 25-hydroxycholecalciferol; chemiluminescence immunoassay “Liaison – 25OH-VitD-Totalassay” (DiaSorin, Italy) or Elecsys “25OH- assay” on COBAS E411 (Roche, Mannheim, Germany), *1,25-(OH)D3* 1,25-dihydroxycholecalciferol; analyzed manually via chromatography and radioimmunosorbent assay (DiaSorin, Italy), *Crea* serum creatinine, *P* serum phosphate, *24 h uCa* 24-h urinary calcium excretion

According to Hesch [20], all patients were classified “asymptomatic” clinically. The patients were neither “minimally symptomatic” (showing arterial hypertension, osteopenia/osteoporosis, hypercalcemic symptoms) nor “symptomatic” (osseous or gastrointestinal manifestation). Particularly, the study patients had no kidney stone disease, nephrocalcinosis, or impaired renal function.

No patient had a pharmacological anamnesis regarding nephrotoxic or lithogenic agents.

Diagnosis of PHPT was based on common laboratory tests [21]. The median age was 58 years (range 33 to 80, interquartile range 17 years [51 to 68]).

Four (10 %) of the 40 patients had a history of kidney stones, two of whom underwent surgical extraction. In the remaining two subjects, the stones passed spontaneously. However, at the time of diagnosis of PHPT (including the preceding 5 years at least) none of those patients was afflicted with kidney stones.

### Imaging studies

US and CT were performed 12 to 48 h prior to parathyroid surgery.

US of the kidneys was performed by a highly experienced radiologist and with a high-end machine (ATL, HDI 5000, Seattle, USA) using a 5 or a 7-MHz curved array transducer. Documentation included multiple anatomic planes, and at least three images in the longitudinal and three in the transverse section, respectively [16].

Multidetector CT (MDCT; Volume zoom, Siemens, Forchheim, GER) of the abdomen was performed with patients in a prone position. The scanning protocol consisted of 80 kV and 100 mAs low-dose scanning using a 4 × 1 mm collimation, 6-mm table feed, 0.5-s rotation time. Reconstruction was obtained in the axial and coronal plane with 1.5-mm slice thickness. No intravenous or oral contrast agent was administered.

### Imaging analysis

The obtained US images were evaluated for the presence and absence of stones, as well as for the number of stones and sizes in the long axis. The stones were defined as hyperechogenicities with dorsal shadowing [16].

MDCT examinations were evaluated on the screen of a PACS System, again for the absence and presence of stones, as well as for the number of stones and sizes in the long axis. The stones were defined as high-attenuating opacities [16].

Two experienced radiologists evaluated the MDCT and US examinations as a team and provided interpretations in unison. Both investigators were blinded to all clinical and biochemical data. In order to prevent bias, MDCT was assessed 1 week after the evaluation of US. The location of each stone was recorded as being in either the right or the left kidney.

In two patients, multiple (uncountable) stones and parenchymal calcifications were found. For size measurements, ten stones of each of those two patients were evaluated which were easily measurable.

### Statistical analysis

Analysis was performed using a statistical software package (SPSS 11, SPSS Inc., Chicago, USA). Comparison of the number of detected stones with MDCT and US was performed with the Wilcoxon signed-rank test. Statistical significance was set at a *p* value of 0.05.

Surgery was performed on all patients and a single parathyroid adenoma was verified histopathologically. By definition, all patients were cured. Cure was documented by normocalcemia 5 years after surgery.

## Results

### Ultrasound

In 4 (10 %) of 40 patients, a total of four kidney stones could be detected. The median size was 6.5 mm, quartiles ranging from 2.25 to 13.75 mm (interquartile range 11.5 mm). Three females and one male were affected. In three cases, the stones were located in the left, in one case in the right kidney.

### Multidetector computed tomography

MDCT identified a total of 41 stones in 15 (38 %) of 40 patients. The size of the stones ranged from 2 to 15 mm with a median size of 3 mm (interquartile range 2.25 mm [2.5–4.75 mm]). Kidney stone formation affected ten females and five males. In 8 (53 %) patients, stones were revealed in both kidneys, in 2 (13 %) in the left and in 5 (33 %) in the right kidney only.

### Ultrasound vs multidetector computed tomography

The number of detected stones was significantly higher with MDCT than with US (41 vs 4, *p* = 0.00124).

In one patient, a kidney stone was suspected with US, but this stone failed to be detected with MDCT. In two subjects, in whom numerous calculi were seen with MDCT, only one was found to have a kidney stone with US, measuring 15 mm in diameter. The remaining calcifications in both kidneys were not seen with US (Table 2).

**Table 2:** Kidney stones detected by ultrasound (US) and multidetector computed tomography (MDCT)

Results		US				Total	
		Positive		Negative			
		*n*	%	*n*	%	*n*	%
MDCT	Positive	3	7.5	12	30.0	15	37.5
	Negative	1	4.0	24	60.0	25	62.5
	Total	4	10.0	36	90.0	40	100.0

*n* = patients; 40 patients were evaluated prospectively

## Discussion

None of the patients had clinical signs of kidney stones upon diagnosis of PHPT or other organ manifestations of classical PHPT [22] and were by definition [1] clinically “asymptomatic.”

Following the current recommendations [23], all patients with biochemically proven PHPT who have no contraindications for a surgical intervention were presented in a multidisciplinary endocrine conference. The risks, benefits, and potential complications of surgery were discussed with the patients and all decided surgery as the treatment of choice.

In this study protocol, MDCT proved significantly superior to US in the detection rate of clinically “silent” kidney stones in patients with initially “asymptomatic” PHPT.

As shown in this study, a number of patients clinically classified “asymptomatic” may have “silent” organ manifestations and therefore be in fact “symptomatic.” In many ways, “symptomatic” patients profit from surgery [2, 9]. In the current guidelines [1], “asymptomatic” patients compose a patients’ group inheriting only a “relative indication” for surgery while in “symptomatic” patients early surgical intervention is recommended [23], so those patient groups need to be discriminated [24].

Therefore, the current guidelines aim to enhance the screening for oblique organ manifestations by recommending renal examinations by ultrasound, the proportion of patients identified with kidney stones might be smaller if ultrasound is used compared to MDCT and consequently, hidden symptoms might be missed. Another study concluded recently [25], that through the use of aforementioned guidelines more surgical candidates are identified. Nevertheless, some patients might persist who do not meet criteria for surgery if only screened with US. We assume that screening for kidney stones by MDCT could direct more patients from this uncertain group towards surgical treatment. Due to the significantly higher number of stone carriers, centers not able or unwilling to perform MDCT to definitively rule out renal involvement should apply a low threshold regarding symptomatology to indicate surgery early because of possibly overlooked kidney stones when applying US only.

The superiority of MDCT in determining the size, number, and the position of kidney stones was convincing in this study, and the possibility of low-dose protocols seems to justify the use of ionizing radiation. As shown, there was a larger number of patients with clinically “silent” kidney stones detected by MDCT compared to US [6, 16], demonstrating the superiority of MDCT and the necessity of an adaption of the current consensus [1].

The performance of MDCT was related to the excellent contrast resolution and discrimination of different attenuation within the kidneys. The lack of acoustic shadowing that may occur with intervening tissue of different acoustic impedance can lead to a miss of stones with US [26]. This could also be the reason for the US diagnosis of kidney stones in one patient that could not be confirmed with MDCT.

MDCT captures a volume that includes the entire kidney, allowing a complete evaluation of the organ. With US, due to potentially overlying bowel gas and patients’ varying body habitus, some areas of the kidney may be hidden [16]. Furthermore, MDCT is less operator-dependent than US which requires skillful radiological expertise [16].

As reported previously, the size of stones seems to influence the detection rate with US. Thus, the median size of stones was 6.5 mm as found with US and 3 mm as detected with MDCT [26]. Most stones missed with US were smaller than 5 mm. Kidney stones with a size of less than 5 mm may pass spontaneously, indicating the limited value of US in follow-up and identification of small stones in known stone formers [16] as well as in distinguishing recurrent from persistent silent kidney stones.

Preoperative radiological assessment is crucial to determine the directions of further postoperative follow-up in patients with “silent” kidney stones [2, 10, 13, 22].

The study documents that MDCT is more sensitive to detect kidney stones than ultrasound.

## Conclusion

Based on low-dose protocols and therefore a lower x-ray exposure, we propose to consider MDCT with its broad availability as the “gold standard” imaging technique for detecting kidney stones in patients with PHPT to identify silent stone carriers in need of surgical treatment, as by this approach patients can be cured who would otherwise develop stones during follow-up [27]. By applying this technique, the very scarce group of patients with “asymptomatic” PHPT may be further diminished. The current guidelines on the management of asymptomatic PHPT [1] should be adapted to this finding. If not used primarily, MDCT should particularly be considered in patients in the border zone of indication for surgery.
